# Academy of Medicine

**Published:** 1880-06

**Authors:** 


### ACADEMY OF MEDICINE.

*Writer's Cramp.*—Dr. Whittaker read a paper on writer's cramp considered an exhaustion of nerve centres induced by excessive use of the same muscles in the same way. The condition was not by any means confined to writers or even the mascles of the hands as it was met with also among telegraph operators, printers, seamstresses, piano and violin players, treadle and treadmill operators, dancers upon upon the tip-toe, etc. In short Benedikt had given the condition the name of "Beschaftigung's Neurosen," avocation or business neuroses.

In the etiology of the affection, the essayist first took up as a very essential factor the neuropathic temperament. Many cases, if not a majority, occurred in individuals of irritable, nervous temperament, subjects themselves or the descendants of parents afflicted with migraine, chorea, epilepsy, paralysis, or some form or other of neurosis. Yet, a certain contingent, if the minority, of cases, occurred independent of any neurosis or any abuse by alcoholism or sexual excess. The prime factor in the etiology of writer's cramp was excessive writing. It was a most important point in diognosis that the first cases of the disease are affected only in writing, but in no other use of the muscles. It was the exercise not of the same muscles, but of the same muscles in the same way, that produces the disease.

Writer's cramp was a disease without a pathology, that is, without a morbid anatomy. The changes induced were "molecular" and not sufficiently gross for ocular demonstration, however aided. It was probable that a defective co-ordination of motor centres was the essence of the pathology of the disease. The essayist here discussed briefly the points favoring this view as opposed to the eccentric or reflex excitation of it.

In symptomatology he adopted the classification of Benedikt into three forms, viz.: the spastic, tremulous and paralytic, of which the spastic was present in more than half the cases.

The diagnosis of the condition was easy enough. It was only necessary to put a pen in the hand of the patient to elicit not only the existence of the condition, but the form of it. The difference between the effects of steel and quill pens was noticed here and the theory that the disease was caused by steel pens refuted by illustrations.

The prognosis of the condition was bad. A few cases recover entirely, but such recovery was the exception and not the rule. Individuals unable to rest the hand seldom recovered entirely, but it would return to the physician again and again, for the rest of their lives. The unfortunate subjects of it who were compelled to force the hand would induce finally a condition of spastic contraction of the arm, the shoulder and side of the trunk, with præcordial anxiety, palpitation and extreme distress, bordering on epilepsy.

The treatment was first, rest. Substitution of the left hand only changed the disease ultimately to the left side.

The various mechanical devices gave only temporary relief, as did also all general treatment by nervines and sedatives, strychnine atropine, etc. Galvanization was the best treatment. Even Duchenne was forced to admit that Faradization was of no avail only in the paralytic and anæsthetic forms of the disease. The galvanization must be gentle and the sessions brief. Galvanization, local hydrotherapy, manuel gymnastics, massage, these were the remedial agents of most efficacy.

Tenotomy had proven useless, but nerve stretching from which so much good has beed accomplished in like conditions elsewhere had not been tried as yet in the relief of writer's cramp.

(The paper will be published in full in a future issue.)

Dr. Zinke remarked that he had never seen or treated the affection under discussion but he would like to ask how the local action of the constant current is explained. He was prompted to ask this question by a belief that the action of galvanization was always general. A man had applied to him a short time ago complaing of heaviness in his limbs. The constant current with the strenth of sixteen cells (one pole on the ball of the foot and the other in the politieal space) was applied, and the effect being *nil*, the number was increased to thirty when the man fainted. The heaviness of the legs was entirely removed on the return of conciousness.

Dr. Ransohoff mentioned a case of a man about 45 years of age who for the last sixteen years had suffered from post spinal sclerosis, the disease coming on very slowly indeed.

A year and a half ago he was attacked with writer's cramp, the pain and contractions commencing in the digits and extending upwards. The patient was engaged at the auditor's office and felt better and could do more work at times when he was very busy. The speaker regarded the as probably due to specific lesion of the cord though large doses of iodide of potassium had failed to give relief. Nerve stretching, he thought, might be of benefit in confirmed writer's cramp, since similar conditions of the nervous system had been relieved by it, and the operation itself not a very hazardous one.

Dr. Buckner remarked that he had about a year ago treated an editress for writer's cramp with decided benefit. The treatment employed was Faradization. R. B. D.

